# Salt stress proteins in plants: An overview

**DOI:** 10.3389/fpls.2022.999058

**Published:** 2022-12-16

**Authors:** Habib-ur-Rehman Athar, Faisal Zulfiqar, Anam Moosa, Muhammad Ashraf, Zafar Ullah Zafar, Lixin Zhang, Nadeem Ahmed, Hazem M. Kalaji, Muhammad Nafees, Mohammad Anwar Hossain, Mohammad Sohidul Islam, Ayman El Sabagh, Kadambot H. M. Siddique

**Affiliations:** ^1^ Institute of Pure and Applied Biology, Bahauddin Zakariya University, Multan, Pakistan; ^2^ College of Life Sciences, Northwest A&F University, Yangling, China; ^3^ Department of Horticultural Sciences, Faculty of Agriculture and Environment, The Islamia University of Bahawalpur, Bahawalpur, Pakistan; ^4^ Department of Plant Pathology, Faculty of Agriculture and Environment, The Islamia University of Bahawalpur, Bahawalpur, Pakistan; ^5^ Institute of Molecular Biology and Biotechnology, The University of Lahore, Lahore, Pakistan; ^6^ Department of Botany, Mohy-ud-Din Islamic University, Nerian Sharif, Pakistan; ^7^ Department of Plant Physiology, Institute of Biology, Warsaw University of Life Sciences SGGW, Warsaw, Poland; ^8^ Department of Genetics and Plant Breeding, Bangladesh Agricultural University, Mymensingh, Bangladesh; ^9^ Department of Agronomy, Hajee Mohammad Danesh Science and Technology University, Dinajpur, Bangladesh; ^10^ Faculty of Agriculture, Department of Field Crops, Siirt University, Siirt, Türkiye; ^11^ Agronomy Department, Faculty of Agriculture, Kafrelsheikh University, Kafrelsheikh, Egypt; ^12^ The UWA Institute of Agriculture, The University of Western Australia, Petrth WA, Australia

**Keywords:** differentially expressed proteins, omics, dehydrins, osmotin, ion transporters, LEA proteins, receptor-like kinases, photosynthesis

## Abstract

Salinity stress is considered the most devastating abiotic stress for crop productivity. Accumulating different types of soluble proteins has evolved as a vital strategy that plays a central regulatory role in the growth and development of plants subjected to salt stress. In the last two decades, efforts have been undertaken to critically examine the genome structure and functions of the transcriptome in plants subjected to salinity stress. Although genomics and transcriptomics studies indicate physiological and biochemical alterations in plants, it do not reflect changes in the amount and type of proteins corresponding to gene expression at the transcriptome level. In addition, proteins are a more reliable determinant of salt tolerance than simple gene expression as they play major roles in shaping physiological traits in salt-tolerant phenotypes. However, little information is available on salt stress-responsive proteins and their possible modes of action in conferring salinity stress tolerance. In addition, a complete proteome profile under normal or stress conditions has not been established yet for any model plant species. Similarly, a complete set of low abundant and key stress regulatory proteins in plants has not been identified. Furthermore, insufficient information on post-translational modifications in salt stress regulatory proteins is available. Therefore, in recent past, studies focused on exploring changes in protein expression under salt stress, which will complement genomic, transcriptomic, and physiological studies in understanding mechanism of salt tolerance in plants. This review focused on recent studies on proteome profiling in plants subjected to salinity stress, and provide synthesis of updated literature about how salinity regulates various salt stress proteins involved in the plant salt tolerance mechanism. This review also highlights the recent reports on regulation of salt stress proteins using transgenic approaches with enhanced salt stress tolerance in crops.

## 1 Introduction

Salinity is a major environmental stressor, particularly in arid and semi-arid regions of the world, causing substantial crop losses (Ashraf and Foolad, 2013; Rengasamy, 2016; Mujeeb-Kazi et al., 2019; Naeem et al., 2020; Zulfiqar and Ashraf, 2021; Ashraf and Munns, 2022). According to some reliable estimates, more than 6% of the world’s land is considered saline (Wicke et al., 2011; Rengasamy, 2016), and of the total irrigated lands, 20% are saline, resulting in estimated agricultural losses of US$27.3 billion annually (Munns et al., 2020a). The extent of saline agricultural land continues to increase mainly due to poor management practices (Chartres and Noble, 2015; Rengasamy, 2016), as do the yield gaps between demand and production. Moreover, agricultural production must increase to feed the rapidly increasing world population estimated to be 9.6 billion by 2050 (Fischer et al., 2009; Arzani and Ashraf, 2016; Fischer et al., 2017; Zafar et al., 2017; Hickey et al., 2019). Efficient utilization of marginal saline lands for crop production could help in meeting demand for crop productivity (Munns and Gilliham, 2015). Another option could be developing salt-tolerant crop plants using conventional breeding or advanced molecular biology-based techniques that can sustain growth on salt-affected soils (Flowers, 2004; Flowers et al., 2010; Ashraf and Foolad, 2013; Flowers et al., 2019). However, success in developing salt-tolerant crop plants has not been particularly successful, mainly due to a poor understanding of salt tolerance mechanisms in plants (Flowers et al., 2010; Mujeeb-Kazi et al., 2019; Ashraf and Munns, 2022). Many scientists thought that salt-tolerant crop plants could be engineered using one gene with one protein acting as a master switch or regulator for a wide variety of physiological and biochemical processes, such as pyrophosphatase (*AVP1*) (Gaxiola et al., 2016), WRKY transcription factors (Jiang et al., 2017; Zhao et al., 2019b), and DREB (Lata and Prasad, 2011). Others thought that specific proteins controlling adaptation or developmental processes to tolerate, avoid or minimize the influence of salt stress in crop plants could be used in traditional breeding or transgenic approaches to increase plant growth and yield on salt-affected land (Munns, 2005; Ashraf et al., 2008; Roy et al., 2014; Dissanayake et al., 2022).

It is widely known that salt stress reduces crop growth through osmotic stress, specific ion toxicity, nutritional imbalance, and impaired hormonal regulation (Munns and Tester, 2008; Zörb et al., 2019). Plants adapt to salinity stress by regulating the uptake and transport of Na^+^ and K^+^ ions, activating enzymes to scavenge reactive oxygen species (ROS), and improving osmotic adjustment (Kalaji and Pietkiewicz, 1993; Ashraf, 2009; Athar and Ashraf, 2009; Munns et al., 2020b). Proteins regulate these processes under normal and salt stress conditions (Li et al., 2017b; Li et al., 2017c; Iqbal et al., 2019), which are responsible for regulating cellular metabolism, organic and inorganic solute transport, water transport, osmoregulation, redox balance, sensing and signalling, hormonal balance, cell division, cell enlargement, and growth and development (Zhang et al., 2012a; Li et al., 2017c). In this review, we discussed the effect of salt stress on total soluble proteins, composition of proteins of different molecular weights, proteins related to various physiological and biochemical processes in halophytes and glycophytes or salt tolerant and salt sensitive cultivars of same species (Bose et al., 2017; Negrão et al., 2017; Pailles et al., 2020). Due to non-availability of sophisticated techniques for proteins, in mid and late 20^th^ century, only total soluble proteins and one-dimensional SDS-PAGE were used. However, in 21^st^ century, 2-D electrophoresis coupled with LC-MS techniques at various platforms (gel-based, with or without label) are being used to describe protein profiles and post-translational modifications. The evolution in use of diverse techniques in proteome profiling under salt has also been reviewed. This review presented recent knowledge about functional specificity of range of promising candidate proteins in response to salt stress, which will help in devising strategies to improve salt tolerance in crops.

## 2 Salt stress proteins: A general account

Several proteins accumulate in plants in response to salinity stress. Plant tissues normally respond to salt stress by degrading proteins or producing abundant salt stress related proteins (Wang et al., 2015b). Moreover, more proteins have been observed in salt-tolerant cultivars than salt-sensitive cultivars of many crops, including sunflower, barley, rice, and wheat and references therein). Thus, the quality and type of proteins may be more important than the quantity of total soluble proteins for salt tolerance (Dissanayake et al., 2022). For example, Ashraf and O'Leary (1999) demonstrated that salt-tolerant wheat cultivar S-24 had fewer total soluble proteins than salt-sensitive Potohar. Moreover, SDS-PAGE revealed that both cultivars had similar protein expression patterns. However, Potohat had lower expression of 29 and 48 kDa proteins than S-24. With advanced molecular biology-based techniques, such as 2-D electrophoresis, MALDI-TOFF, and LC-MS, complete protein profiling can be performed in salt-stressed plants. Bioinformatics tools can further help identify specific salt-stress proteins. Changes in protein expression under salt stress are mainly associated with several biological functions and post-transcriptional and post-translational changes (Chiconato et al., 2021; Jha, 2022). These proteins are directly involved in determining new phenotypes that can adapt to salt-stressed environments by contributing to vital metabolic processes. Thus, the contribution of specific proteins is more important for salt tolerance mechanisms than protein quantity.

**Figure 1:**
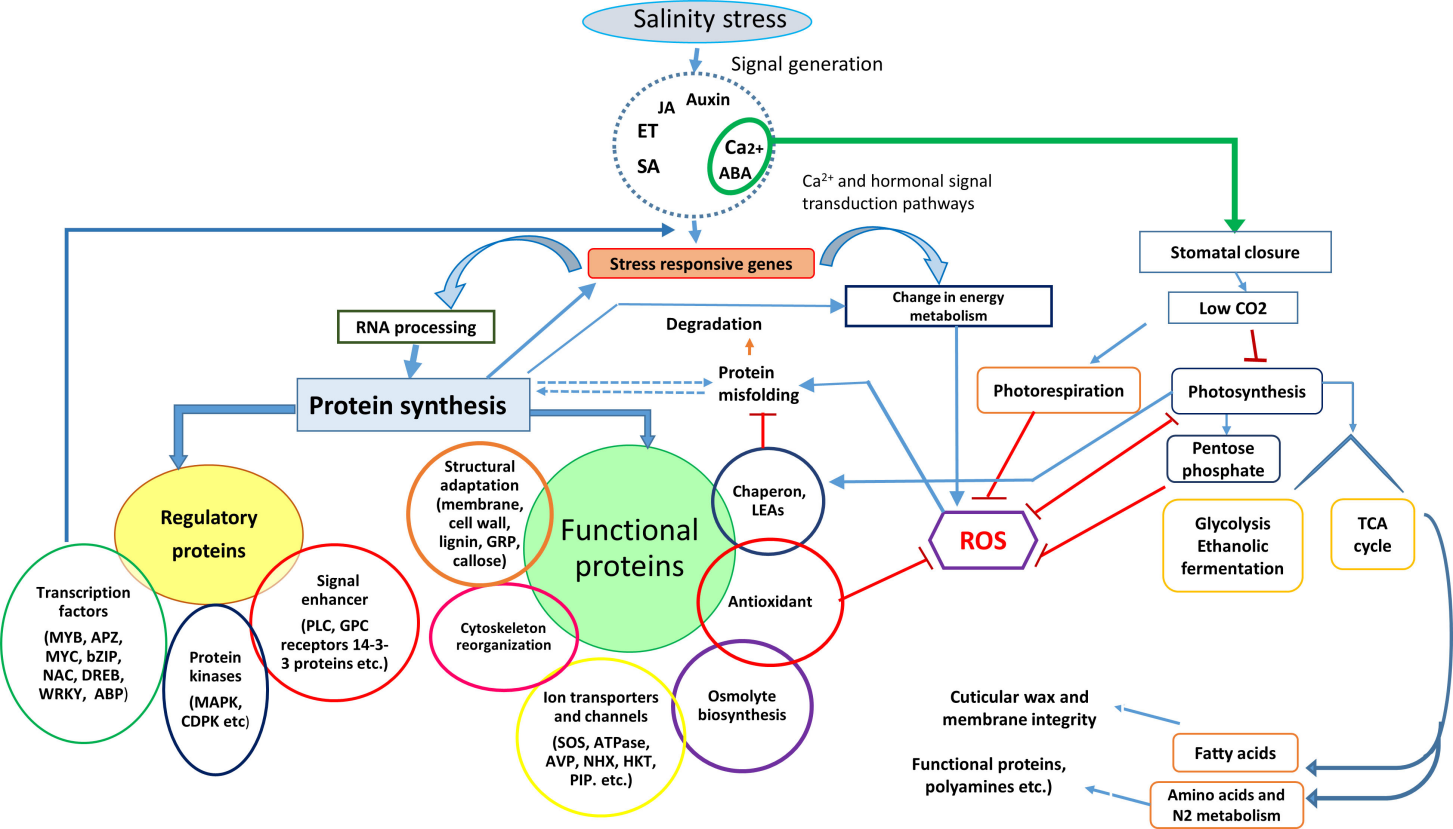
Schematic representation of physiological and biochemical events in plants exposed to salt stress over time and the salt stress proteins expressed in different plant parts to execute the salinity stress tolerance mechanism.

Based on their functions in salinity or general stress tolerance, these proteins are grouped as salt stress and stress-associated proteins (**Figure 1**). Salt stress proteins accumulate only in response to salt stress, whereas stress-associated proteins accumulate under any stress.

It is well-known that salt stress has a similar osmotic effect to drought stress in plants. Soluble proteins that accumulate in plants under salt stress play a key role in osmoregulation, including osmotin in *Mesembryanthemum crystallinum* (Thomas and Bohnert, 1993; Qun et al., 2017) and tomato (Goel et al., 2010), salt shock protein in sugar cane (Gomathi and Vasantha, 2006), germin in barley (Hurkman et al., 1991), and osmotin-like proteins in sesame (Kosová et al., 2013; Chowdhury et al., 2017; Qun et al., 2017). Some proteins are enzyme complexes responsible for regulating the biosynthesis of soluble sugars, which play a role in osmoregulation under salt stress (Zhao et al., 2019a). For example, salt stress reduced fructose-2,6-bisphosphatase levels in salt-stressed rice leaves, increasing sucrose accumulation, which regulated cell osmolarity and thus improved salt tolerance (Udomchalothorn et al., 2009). While many proteins are not involved in osmoregulation, they protect cell membranes, structural proteins, and enzymes from Na^+^ toxicity and Na^+^-induced dehydration, e.g., heat shock proteins (HSPs) and late embryogenesis abundant (LEA) proteins (Chourey et al., 2003; Park et al., 2005; Song and Ahn, 2011; Derevyanchuk et al., 2015). Recently, Rodriguez et al. (2021) reported that salt stress-induced post-translational modifications in 245 proteins by phosphorylation and in 35 proteins by lysine acetylation played a significant role in salt tolerance. They further added that changes in abundance of 107 proteins involved in various metabolic pathways such as osmo-protection, sulfur metabolism of *Arabidopsis thaliana* could be candidate proteins for salt tolerance. Similarly, maintenance of ion homeostasis is a key component of salt tolerance, regulated by several membrane proteins responsible for ion transport, including K^+^ and Na^+^ channels and pumps (Huang et al., 2008; Ji et al., 2013; Huang et al., 2020), and transport-facilitating cytosolic proteins involved in intra-cellular communication, such as CIPK6 (Roy et al., 2013). Such proteins increase when plants are exposed to salt stress (Ji et al., 2013; Qun et al., 2017; Luo et al., 2018; Ma et al., 2019).

Some proteins play a dynamic role in sensing and conferring salt tolerance, including receptor-like protein kinases (RLKs) (Yang et al., 2010; Ye et al., 2017) and primary cell wall ‘sensors’ such as caffeoyl-CoA *O*-methyltransferase (COMT) (Wang et al., 2019b). While working with salt-tolerant and salt-sensitive rice cultivars (FL530-IL, KDML105), Saeng-Ngam et al. (2012) found 24 and seven-fold increase in transcript and protein abundance of calmodulin protein (*OsCam1-1*) within 30 minutes. In addition, over-expression of calmodulin protein in *OsCam1-1* rice transgenic plants showed a greater salt tolerance, which indicated that *OsCam1-1* act as salt sensor protein.

Different cellular organelles possess different types of proteins that play various roles in metabolism (Jacoby et al., 2011; Bose et al., 2017; Luo et al., 2017; Goussi et al., 2021). For example, chloroplasts contain a myriad of proteins (e.g., thylakoidal photosystem II, cytochrome b6-f complex, iron-sulfur proteins, oxygen-evolving enhancer proteins (OEE), photosystems-I, ATP synthase) and stromal proteins (e.g., ribulose-1,5-bisphosphate carboxylase (rubisco) and fructose-bisphosphate aldolase (pFBA)) (Meng et al., 2016a; Xu et al., 2016; Yousuf et al., 2016; Suo et al., 2017) that are integral parts of key metabolic processes. Similarly, various proteins have been identified in mitochondria under salt stress, including Mn-superoxide dismutase (Mn-SOD) and oxidase (AOX) (Jacoby et al., 2011; Wojtyla et al., 2013; Che-Othman et al., 2017; Goussi et al., 2021). Likewise, vacuoles play a vital role in maintaining ion homeostasis, osmotic adjustment, and cell turgor under salt stress (Barkla et al., 2009; Barkla et al., 2013a; Barkla et al., 2013b). Barkla et al. (2009) reported that salt stress altered tonoplast-associated glycolytic enzymes, such as aldolase (ALDO), enolase (ENO), V-ATPase, and H-ATPase *in Mesembryanthemum crystallinum* and upregulated H^+^-pump activity to maintain ion homeostasis. The endoplasmic reticulum (ER) is involved in mediating salt tolerance through calcium ion (Ca^2+^) homeostasis, lipid metabolism and synthesis, and the folding, assembly, transport, and modification of secretory and transmembrane proteins responsible for sensing environmental cues (Liu and Li, 2019; Park and Park, 2019; Yu et al., 2019). For example, Wang et al. (2011) reported that salt stress accumulated unfolded proteins in ER in Arabidopsis plants. Similarly, Zhan et al. (2019) found 317 of 5,774 proteins were differentially expressed in salt stressed okra (*Abelmoschus esculentus* L.), with most associated with ‘response to stress’ and ‘protein processing in the ER’. Zhu et al. (2019) demonstrated that an ER protein, ZmSep15‐like‐2, which interacts with ZmUGGT1 (UDP‐glucose: glycoprotein glucosyltransferase1, UGGT1) induced salt tolerance in *Arabidopsis thaliana* by mitigating salt-induced oxidative stress. Moreover, the authors revealed that interrupting this interaction caused salt sensitivity in Arabidopsis plants. Thus, each cellular compartment comprises specific proteins that undergo differential regulation on exposure to salt stress.

However, a protein isolated from one species may have different functional capabilities than that in other plant species. For example, significant genetic diversity exists in the amino acid sequence of selective pore-loops in the HKT1 gene family with variable capability to induce salt tolerance in plants (Shohan et al., 2019; Huang et al., 2020; Dissanayake et al., 2022). Protein profiling and their characterization under salt stress with different physio-biochemical functions will provide more information about candidate proteins (**Figure 1**). Next, we discuss different types of proteins and their physio-biochemical functions under salt stress.

## 3 How stress proteins enable plants to resist salt stress

Salt stress affects plant growth and development, and plants have evolved intricate mechanisms to cope with these environmental cues. Successfully executing plant stress tolerance processes requires coordinated activity between each component (Morton et al., 2019; Pailles et al., 2020; Nguyen et al., 2022). For example, salt stress causes ROS generation and changes the cellular redox balance, affecting cytosolic Ca^2+^ by activating calcium channels, an activity connected to Ca^2+^-dependent sensing and signalling pathways. Plants convert this salt stress signal by activating another group of salt stress proteins (protein kinases and phosphatases) to trigger target proteins (transcription factors or switches) in different metabolic networks, such as biosynthetic enzymes for different phytohormones, enzymes for nutrient metabolism (e.g., N metabolism), enzymes and cell wall proteins for cell division and enlargement, enzymes for carbohydrate metabolism in photosynthesis and respiration, and proteins for regulating plant growth and development, such as transcription factors in the MADS-box class for flowering (Yokthongwattana et al., 2012; Long et al., 2016; Tan et al., 2016; Jusovic et al., 2018; Nguyen et al., 2022). In the cross-talk between different signaling networks, various salt-stress proteins regulate osmoregulation and ion transport and compartmentalize toxic ions in the vacuole. Such activities require the coordinated activity of vesicles and proteins trafficking in different cellular organelles (Nam et al., 2012; Wang et al., 2015b; Zhao et al., 2018; Gan et al., 2021). Thus, plants likely express specific proteins to perform particular functions (**Figure 1**). The next section discusses the different types of salt stress proteins.

### 3.1 Salt stress proteins involved in different metabolisms

Plants reprogram their metabolism to enhance salinity tolerance (Barajas-Lopez et al., 2018; Jardak et al., 2021) by using specific pathways that are salt stress imprints (Schwachtje et al., 2019; Zhang et al., 2022). For example, two inbred maize lines had 57 differentially expressed proteins under salt stress, related to energy metabolism, carbohydrate metabolism, antioxidant system, secondary metabolite biosynthesis, protein refolding, protein translation, and transcriptional regulation. This specific network of metabolic pathways can be used as salt stress imprints (Cheng et al., 2014). Such changes in metabolism are due to salt stress sensor proteins and metabolic biosynthesis enzymes directly involved in the acquisition of salinity tolerance (Hey et al., 2009; Barajas-Lopez et al., 2018; Yang et al., 2019; Jha et al., 2022).

#### 3.1.1 Salt stress proteins involved in nitrogen metabolism

Several studies have demonstrated that SnRK kinases sense the cellular energy state, activating the biosynthesis of proline and soluble sugars such as sucrose, trehalose, and fructans that can stabilize cellular structures under salt stress (Figueroa and Lunn, 2016; Baena-González and Hanson, 2017; Barajas-Lopez et al., 2018; Belghith et al., 2022). Proline accumulation in salt-stressed plants is mainly associated with *de novo* synthesis from glutamate and N metabolism (Zulfiqar and Ashraf, 2022), e.g., in watermelon (Yang et al., 2013) and cucumber (Shao et al., 2015). While plants can uptake N as nitrate (NO_3_^–^) or ammonium (NH_4_^+^), nitrates are the main source of nitrogen for agricultural productivity (Taiz et al., 2015). Therefore, nitrate uptake by nitrate transporters and its transport are sensitive to salinity stress (Flores et al., 2000; Flores et al., 2004). Similarly, NH_4_^+^ assimilation is affected by salt stress, affecting key N metabolic pathways such as the biosynthesis of amino acids, protein, and secondary metabolites (Popova et al., 2002; Debouba et al., 2006). The assimilated N from NO_3_^–^or NH_4_^+^ sources is invested in structural proteins related to plant photosynthetic machinery. All N uptake, transport, and metabolism processes impact photosynthetic activity and plant growth under abiotic stress, including salt stress (Chen et al., 2012). For example, while assessing the impact of NO_3_^–^ and NH_4_^+^ sources on sunflower growth and photosynthetic activity, Ashraf (1999) found that NH_4_^+^-N inhibited the growth of non-stressed sunflower plants, while NO_3_^–^N had a greater inhibitory effect on salt-stressed plants; these inhibitory effects were associated with an inhibited photosynthetic rate, suggesting that N metabolism is linked to C metabolism. Similarly, Tian et al. (2022) found the similar findings with a leguminous tree species *Sophora japonica*. However, they reasoned that over-expression of N-assimilation enzymes like nitrate reductase, glutamine synthase, nitrate transporters etc. While assessing effects of salt stress on proteome changes in halophyte *Cakile maritima* Belghith et al. (2022) found changes in protein expression related to N-metabolism played a major role in degree of salt tolerance. Thus, changes in the proteins associated with N and C metabolism may alter the degree of salt tolerance in plants.

Plants comprise low- and high-affinity nitrate transporter families, namely NRT1 and NRT2, which sense soil nitrate concentrations *via* a phosphorylation dephosphorylation mechanism (Tsay et al., 2007). Decreased expression or disruption in their function due to abiotic stress can reduce nitrate uptake in plants (Li et al., 2007). Nitrates taken up into roots are transported *via* two long-distance nitrate transporters (NRT1.5 and NRT1.8) to leaf mesophyll cells and assimilated in chloroplasts (Li et al., 2010). The NRT1.5 nitrate transporter is expressed in the root pericycle cells, while NRT1.8 is expressed mainly in xylem parenchyma cells. However, they both transport nitrates from roots to shoots (Lin et al., 2008; Li et al., 2010; Meng et al., 2016b; Ashraf et al., 2018). Nitrate assimilation is an energy-intensive process. Plants transport nitrates from roots to shoots, where photosynthetic reductants such as ATP, NADPH, and Fd are involved in nitrate assimilation; thus, shoot nitrate assimilation is more efficient than root nitrate assimilation (Taiz et al., 2015). Several studies have shown that abiotic stresses, including salt stress, reallocate nitrates to roots by downregulating NRT1.5 and NRT1.8 transporters (Li et al., 2010; Chen et al., 2012; Ashraf et al., 2018). In addition, functional disruption of NRT1.5 in *nrt1.5* mutants of *Arabidopsis thaliana* plants changed the expression of salt-stressed marker genes, particularly those regulating Na^+^ distribution (e.g., *HKT1* and *SOS1*) and osmolyte synthesis (e.g., *P5CS1* and *AtPCS1*) (Chen et al., 2012). Thus, changes in the shoot and root N assimilation patterns, due to the availability of different N sources or salt stress, change the degree of salt tolerance in plants.

#### 3.1.2 Salt stress proteins involved in photosynthesis and carbohydrate metabolism

Carbon or carbohydrate metabolism occurs in the chloroplast, mitochondria, and cytosol of plant cells *via* the Calvin cycle, pentose phosphate pathway, glycolysis, and Krebs cycle (Barkla et al., 2013b; Taiz et al., 2015). Therefore, salt stress downregulates or upregulates several proteins associated with these metabolic pathways, as observed in rice (Nam et al., 2012; Liu et al., 2014), sorghum (Ngara et al., 2012), alfalfa (Xiong et al., 2017), canola (Iqbal et al., 2019), chickpea (Arefian et al., 2019) and *Eutrema salsugineum* (Goussi et al., 2021). Several studies have reported that salt stress reduces proteins related to chlorophyll biosynthesis, but increases thylakoidal proteins related to the light reaction (Barkla et al., 2013b; and references there in). Similarly, Li et al. (2017c) reported that the expression of thylakoidal proteins (Psb27, PsaO, PetC, and LHCs) increased in the leaves of salt-stressed *Carex rigescens*, contributing to salt tolerance. Arefian et al. (2019) reported the upregulation of chlorophyll a/b binding proteins and OEE proteins in chickpea under salt stress. Liu et al. (2019) reported overexpression of the D2 protein of photosystem II and chlorophyll a/b binding protein in the Chinese herbal medicine *Spica prunellae* under salt stress. Ji et al. (2019) found three chlorophyll a/b binding proteins of 13 photosynthesis-related proteins overexpressed in banana plants (*Musa paradisiaca*) under salt stress. In contrast, Xiong et al. (2017) reported the downregulation of thylakoidal proteins carrying out light reactions, such as chlorophyll a/b proteins, cytochrome b6-f complex, and OEE proteins, salt-stressed alfalfa. While working *Eutrema salsugineum* Goussi et al. (2021) found changes in abundance of 58 proteins related with thylakoidal reactions. The major changes occurred in core protein of PSII (D1, D2, CP43, CP47, PsbE and PsbH), whereas changes in sub-units of oxygen evolving complex and cytochrome b6-f complex remained constant. The downregulation of chlorophyll biosynthesis proteins and upregulation of chlorophyll a/b binding proteins, as part of the light harvesting complex of photosystem II, help adjust antenna size of PSII and light absorption to avoid ROS generation. Downregulation of proteins such as cytochrome b6-f complex and photosystem I regulate electron transport to reduce electron transfer to oxygen, thus avoiding ROS generation. Moreover, overexpression of the D2 protein of PSII, as part of the PSII repair cycle, helped plants to maintain PSII functionality under salt stress.

CO_2_ fixation into sugars occurs *via* the RuBisCo enzyme in the Calvin cycle. Salt stress reduces plant growth by affecting the rate and amount of CO_2_ fixation by RuBisCo and other enzymes in the Calvin cycle. For example, Wang et al. (2015a) reported that salt stress upregulated the protein expression of RuBisCo small subunit 1, RuBisCo large subunit 1, and beta-carbonic anhydrase in the halophyte *Halogeton glomeratus*, but downregulated the expression of Calvin cycle related enzymes, such as phospho-ribulokinase, putative transketolase, sedo-heptulose-1,7 bisphosphatase, and RuBisCo activase. In contrast, Kamal et al. (2012) reported that salt stress inhibited the expression of RuBisCo in wheat. Fan et al. (2011) reported that the abundance of RuBisCo in halophytic *Salicornia europaea* was similar under non-saline and moderately saline conditions but decreased under high salt stress (200 mM NaCl). Moreover, at the high salt level, the abundance of RuBisCo activase increased, helping convert RuBisCo from inactive to active form, thus increasing the CO_2_ fixation rate. The same authors found that moderate salt stress increased the abundance of proteins related to Calvin cycle enzymes (e.g., phosphoglycerate kinase, glyceraldehyde-3-phosphate dehydrogenase subunit B, sedo-heptulose, and 1-7 bisphosphatase, and transketolase), whereas high salt stress decreased these proteins. Ji et al. (2019) reported 13 photosynthetic proteins, including RuBisCo and ribose-5-phosphate isomerase, overexpressed in *Musa paradisiaca* subjected to moderate salt stress (60 mM NaCl). In a comparative proteome analysis, Pang et al. (2010) reported that RuBisCo, RuBisCo activase, and other Calvin cycle enzymes were downregulated in glycophyte *Arabidopsis thaliana* but upregulated in halophyte *Thellungiella halophyla* under salt stress, and suggested that these differences were mainly due to the maintenance of RuBisCo activity by RuBisCo activase. While working with castor bean plants (*Ricinus communis*) Wang et al. (2021) found a total of 15 alkali-responsive proteins in the leaves that are involved in CO_2_ fixation. It can be inferred that the extent of salt stress effects on photosynthetic activity of glycophytic and halophytic plants mainly depends on their ability to balance the reducing power generation through the light reaction and its use in CO_2_ fixation *via* the Calvin cycle. In addition, the ability of plant species to manage excess reducing power production relates to their degree of salt tolerance. The differential expression of photosynthetic proteins under salt stress ranges from 17–38% of the total, and depends on the species involved and intensity of salt stress. In most studies, proteomic analysis is undertaken on 2–3-week-old seedlings. Some plant species reach their maximal photosynthetic rate at three to six weeks of vegetative growth; hence, comparative proteome analyses at different developmental stages are required to better understand the changes in photosynthetic proteins under salt stress.

#### 3.1.3 Salt stress proteins involved in cellular respiration

Efficient energy production is pivotal for plant adaptation to salt stress as various adaptive responses are energy-requiring processes, e.g., biosynthesis of organic osmolytes for osmotic adjustments, activation of antioxidant enzymes for ROS scavenging, compartmentation of toxic ions into vacuoles, and transport of metabolites, hormones, and proteins (Barkla et al., 2013a; Kosova et al., 2013; Li et al., 2015; Li et al., 2017b; Li et al., 2017c; Luo et al., 2018; Ji et al., 2019; Zhan et al., 2019). In addition, plant growth under salt stress requires more ATP than no stress. Cellular respiration is responsible for the regulated release of energy from carbohydrates in different cellular compartments, and it occurs in the cytosol (glycolysis, pentose phosphate pathway) and mitochondria (Krebs cycle and oxidative phosphorylation) (Barkla et al., 2013a; Kosova et al., 2013). While working with castor bean plants (*Ricinus communis*) Wang et al. (2021) found a total of 25 alkali-responsive proteins in the roots that are involved in energy metabolism in cytosol and mitochondria. Likewise, Yildiz and Terzi (2021) found increase in abundance of 19 proteins that are related with glycolysis and TCA in a xero-halophyte *Salsa crassa*. Over-expression of mitochondrial proteins and energy metabolism have also been observed in glycophyte rice (López-Cristoffanini et al., 2021). Salt stress upregulates several glycolysis enzymes, including phospho-fructokinase, enolase, and glyceraldehyde-3-phosphate dehydrogenase, to generate energy for repairing salt-induced damage or adapting to salt stress (Li et al., 2015; Liu et al., 2019). For example, salt stress increased the protein abundance of alcohol dehydrogenase in soybean genotypes (Ma et al., 2012). Alcohol dehydrogenase reduces acetaldehyde to ethanol through NAD^+^ re-oxidation and is essential for glycolysis (Ma et al., 2012; Taiz et al., 2015). The energy status of a cell is also related to the activity of enzymes in the Krebs cycle. For example, Jiang et al. (2007) found that salt stress decreased the expression and activity of malate dehydrogenase in Arabidopsis roots. Similarly, Liu et al. (2019) reported the upregulation and increased activity of glycolytic enzymes and Krebs cycle enzymes in salt-tolerant *Spica prunellae*. In contrast, Li et al. (2015) reported that salt stress downregulated proteins associated with the glycolytic pathway and Krebs cycle in cotton seedlings. Salt stress also affects several ATP-generating enzymes, such as ATP synthase, which was downregulated in glycophytic banana plants exposed to 60 mM NaCl (Ji et al., 2019), but upregulated in halophytic *Halogeton glomeratus* (Wang et al., 2015a), *Salicornia europaea* (Wang et al., 2009), and *Aeluropis lagopoides* (Sobhanian et al., 2010). Moreover, Wang et al. (2015a) reported that salt stress upregulated NAD(P)H-quinone oxido-reductase, triose-phosphate isomerase, and lactoyl-glutathione lyase-like in *H. glomeratus*, increasing ATP generation in cells and thus improving salt tolerance. In another study, salt tolerance in canola genotypes was related to an over-accumulation of proteins related to glycolysis (phosphoglycerate kinase 3, fructose-bisphosphate aldolase, and glyceraldehyde-3-phosphate dehydrogenase) and energy metabolism (ATP synthase subunit B) (Kholghi et al., 2019). Plants sensitive to salt stress cannot maintain cellular respiration and produce lower amount of energy than required for salt stress adaptation.

Plant metabolic and biosynthetic pathways are linked to mitochondrial activity and oxidative phosphorylation. Moreover, organic acids and their intermediates are shuttled to different cellular compartments, such as the photorespiratory pathway (Taiz et al., 2015). Salt stress imposes deleterious effects on mitochondrial processes, including oxidative phosphorylation, metabolite transport carrier proteins, and antioxidant enzymes. Some reports have demonstrated that mitochondrial respiration is involved in mediating salt stress tolerance in different plants, e.g., wheat (Jacoby et al., 2010), barley (Widodo et al., 2009), *Rubinia pseudoacacia* (Luo et al., 2017), and *Arabidopsis thaliana* (Smith et al., 2009). Mitochondrial activity thus plays a pivotal role in plant adaptive responses to salt stress (Jacoby et al., 2011; Che-Othman et al., 2017). For example, in wheat, Jacoby et al. (2010) found that salt stress caused a marked over-accumulation of proteins responsible for ROS scavenging, including Mn-SOD, cysteine synthase, nucleotide di-phosphate kinase, and voltage-dependent anion channels. Moreover, these proteins are differentially overexpressed in the salt-tolerant wheat cultivar Wyalkatchem and salt-sensitive wheat cultivar Janz. Similarly, Chen et al. (2009) found that salt stress upregulated glycoside hydrolase, mitochondrial HSP 70, and Cu/Zn-superoxide dismutase and downregulated ATP synthase beta-subunit and cytochrome c oxidase subunit 6b.

Various studies have demonstrated that mitochondrial proteins are salt stress responsive proteins linked to Krebs cycle related proteins, metabolism-related proteins, membrane transport proteins, antioxidant defense related proteins, and HSPs. Thus, salt stress differentially regulates mitochondrial proteins, convincing evidence that an increase in mitochondrial antioxidant defense proteins plays a significant role in plant salt tolerance.

### 3.2 Stress proteins involved in water and ion transport

Biological membranes are barriers to water and ion transport in plant cells. Sustained plant growth requires tight control of ions, particularly Na^+^ uptake, its redistribution among different organs, and accumulation into cell vacuoles, especially under saline stress (Munns and Tester, 2008). While it is well-known that Na^+^ exclusion from shoots or leaves and maintenance of K^+^ homeostasis are characteristics of salt tolerance in several crop species (Naeem et al., 2020; Chen et al., 2021), Cl^–^ exclusion may be another important component for protecting photosynthetic tissues (Li et al., 2017a). Some proteins play an integral role in Cl^–^, Na^+^, and K^+^ transport from roots to shoots and within cells (cytosol to vacuoles) (Roy et al., 2014; Li et al., 2017a; Munns et al., 2020a). Regulation of Na^+^ transport is mediated by sodium loading and unloading in the xylem of parenchyma cells, sodium exclusion from roots to soil, and sodium compartmentation in vacuoles (Roy et al., 2014; Ismail and Horie, 2017; Li et al., 2017a; Huang et al., 2020; Munns et al., 2020a).

#### 3.2.1 Salt stress proteins involved in Na^+^ transport and exclusion

**Figure 2:**
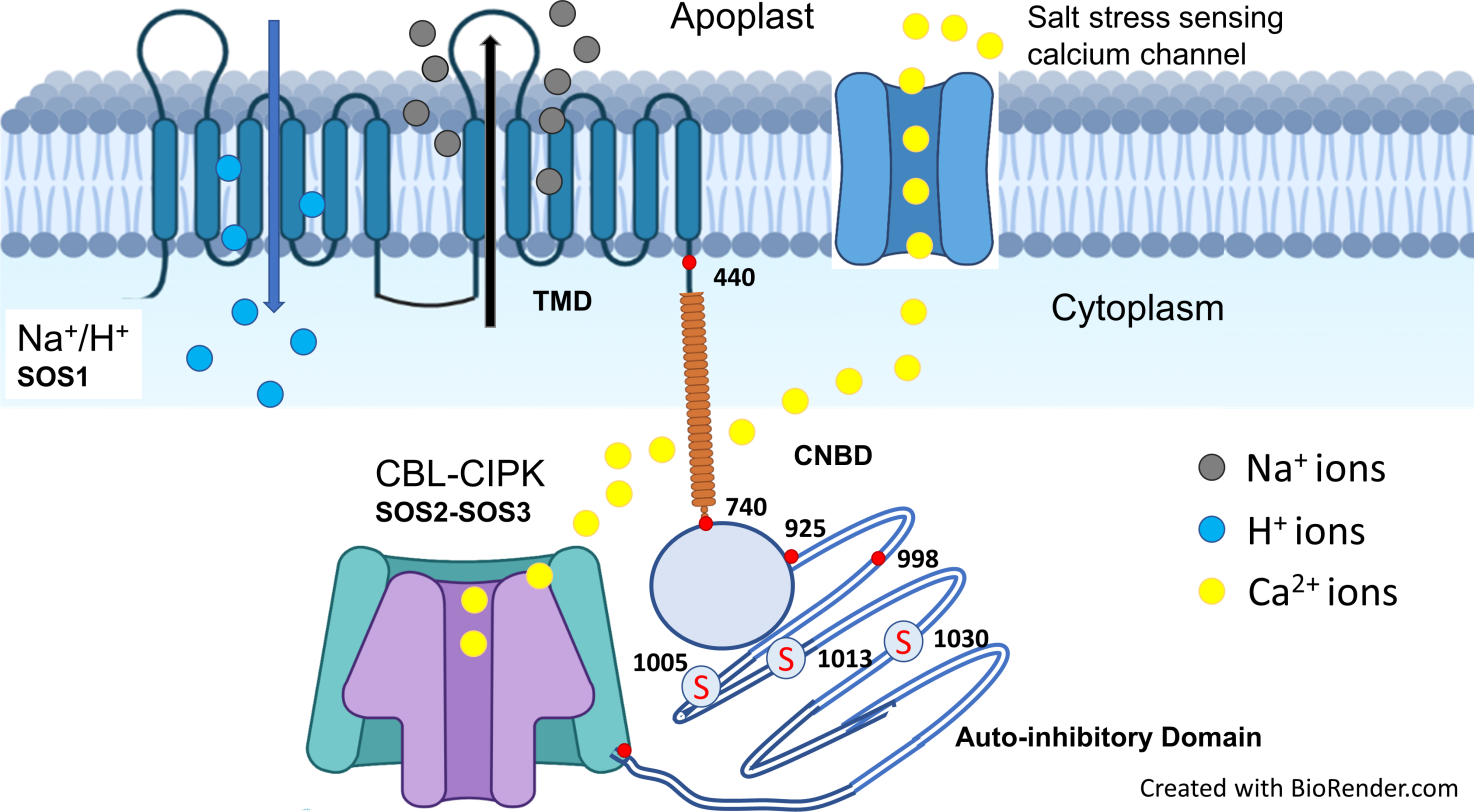
Salt stress is sensed by calcium channel that increased the cytosolic calcium. Increase in cytosolic calcium activates CBL (SOS3) which interacts with serine-threonine protein kinase CIPK (SOS2) that phosphorylates at serine residues of cytosolic C-terminal domain of SOS1 (Na^+^/H^+^ antiporter). Upon activation, SOS1 exclude the Na^+^ using pH gradient as a source of energy. Structure of Na^+^/H^+^ antiporter with 12 transmembrane domains (TMD; 1-440), cytosolic functional domain (CNBD; 741-925) and C-terminal auto-inhibitory domain (998-1146). The red mark at the end of C-terminus represents DSPS, a conservative phosphorylation site recognized by the calcineurin B-like protein (CBL)-CIPK protein kinase complexes. A serine threonine protein kinase (CIPK; SOS2) phosphorylates on three serine sites at the C-terminal end and highlighted as “S” in red with circle (Adapted from Ismail and Horie, 2017; Shohan et al., 2019; Mansour and Hassan, 2022).

Sodium exclusion from roots is undertaken mainly by the plasma membrane Na^+^/H^+^ antiporter -a well-known gene *SOS1*. Silencing or mutation of this gene increased salt sensitivity in different crop species, indicating its putative role in Na^+^ exclusion and hence salt tolerance, e.g., rice (Martinez-Atienza et al., 2007), tomato (Olías et al., 2009), wheat (Cuin et al., 2011), Chrysanthemum (Gao et al., 2016), and Arabidopsis (Feki et al., 2014a). Structural and functional analyses of Na^+^/H^+^ antiporters or *SOS1* gene suggest that it is a membrane protein of 127 kDa with 12 trans-membrane domains, cytosolic N-terminal end, and long cytosolic C-terminal hydrophilic domain (Shi et al., 2000). The C-terminal long tail contains the phosphorylation site, autoinhibitory domain, and cyclic nucleotide-binding domains (**Figure 2**) (Feki et al., 2011). Na^+^/H^+^ antiporter activation requires inhibition of the autoinhibitory domain and phosphorylation at the C-terminal (Feki et al., 2011). The AtNHX8 protein, similar to *SOS1*, lacks the C-terminal end and did not increase salt tolerance in transgenic *Arabidopsis thaliana* plants (An et al., 2007). However, deletion of the autoinhibitory domain from the Na^+^/H^+^ antiporter (SOS1) produced a constitutively active form of the antiporter, increased water and K^+^ uptake, reduced Na^+^ uptake, and thus increased salt tolerance in *Arabidopsis thaliana* (Feki et al., 2014b) and tobacco (Zhou et al., 2016). Phosphorylation of Na^+^/H^+^ antiporters (SOS1) is regulated by two other protein kinase complexes, CIPK24 (SOS2) and CBL4 (SOS3) (Quintero et al., 2011). SOS2 (CIPK) is a kinase of serine-threonine kinase type belonging to the SnRK3 family (Barajas-Lopez et al., 2018), while CBL is a member of the calcium-binding proteins that sense hyper-cytosolic Ca^2+^ (Roy et al., 2013; Roy et al., 2014). Mutations in both genes cause hypersensitivity to salt stress, indicating their potential role in Na^+^ exclusion and salt tolerance (Ismail and Horie, 2017). However, Na^+^ exclusion is an energy-consuming process coupled with H^+^-ATPase activity. For example, salt-tolerant wheat cultivars (Kharchia-65 and S-24) had greater H^+^-ATPase expression and activity in epidermal roots cells than the salt-sensitive cultivar (Potohar), which was associated with Na^+^ exclusion (Ayala et al., 1997; Cuin et al., 2011).

Compartmentation of Na^+^ in the vacuole by Na^+^/H^+^ antiporters localized on the vacuolar membrane helps maintain low Na^+^ in the cytosol (Apse et al., 1999; Apse et al., 2003; Apse and Blumwald, 2007; El Mahi et al., 2019). Overexpression of vacuolar Na^+^/H^+^ antiporters increased salt tolerance in wheat (Cuin et al., 2011), *Vigna unguiculata* (L.) Walp. (Mishra et al., 2014), apple (Li et al., 2013), and several other crops (Roy et al., 2014). Several studies have shown that vacuolar Na^+^/H^+^ exhibited K^+^/H^+^ exchange activity in addition to Na^+^/H^+^ activity (Leidi et al., 2010; Bassil et al., 2011; Bassil et al., 2012; Ismail and Horie, 2017), supported by vacuolar H^+^-ATPases and pyrophosphatases and important for salt tolerance (Shen et al., 2015; Gaxiola et al., 2016). Plasma membrane H^+^-ATPase pumps in the tonoplast pump H^+^ into the vacuoles, providing adequate protons for Na^+^/H^+^ antiporters, as evidenced by overexpressing V-PPases related to the pyrophosphate-driven proton pump (NbVHP) in *Nicotiana benthamiana* leaves showing improved salinity tolerance (Graus et al., 2018).

#### 3.2.2 Salt stress proteins involved in K^+^ transport and maintenance of the K^+^/Na^+^ ratio

**Figure 3:**
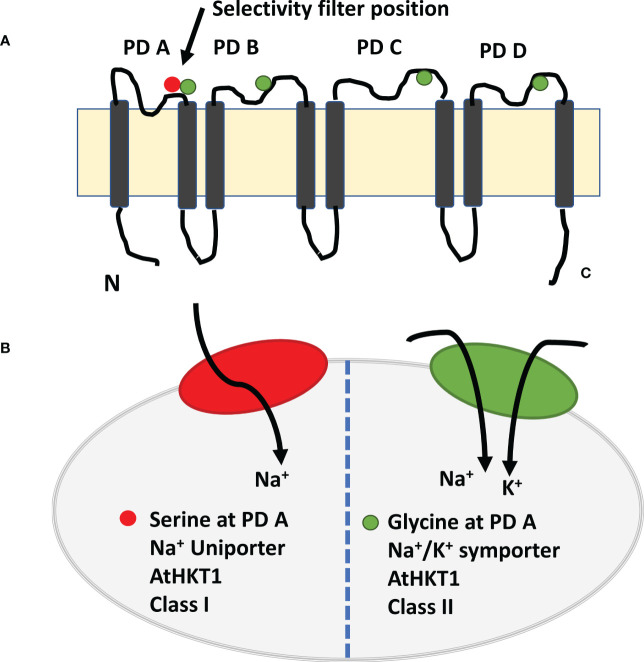
Model of high affinity potassium channel HKT1 with eight transmembrane domain and four pore domains. **(A)** The selective pore or selectivity filter in class 1 has Ser-Gly-Gly-Gly, whereas members of class 2 have selective pore with Gly-Gly-Gly-Gly. Four amino acids are placed on top of the four short loops or PD loop and make it as selectivity filter. The selective filters transport the Na^+^ ion selectively from xylem vessel to xylem parenchyma cells as xylem-Na-unloading process. **(B)**. Class 1 HKT channel act as Na^+^ uniporter only, whereas Class 2 HKT channel as Na^+^ and K^+^ channel (Adapted from Ismail and Horie, 2017; Naeem et al., 2020; Mansour and Hassan, 2022).

The high-affinity potassium transporter (*HKTs*) gene family and proteins of the salt overly sensitive pathway have a potential role in regulating Na^+^ transport and K^+^ homeostasis (Roy et al., 2014). Of the two sub-families of HKTs, HKT1 members have great potential to improve salt tolerance (Ren et al., 2005; Rus et al., 2006; Ahmadi et al., 2011; Wang et al., 2015c; Ali et al., 2016; Jaime-Pérez et al., 2017; Zhang et al., 2017; Xu et al., 2018; Wang et al., 2019a). Members of the HKT1 family are Na^+^ transporters (channel-like Na^+^ uniport) are present on the plasma membrane of root xylem parenchyma cells. They retrieve Na^+^ ions from the xylem stream to reduce sodium transport to shoots (Roy et al., 2014; Tounsi et al., 2016; Shohan et al., 2019). The sodium selectivity over potassium in HTK1s is due to the presence of a serine residue at the first pore-loop domain (Davenport et al., 2007; Ali et al., 2016; Ismail and Horie, 2017). In contrast, members of the HKT2 sub-family have a glycine residue at the first pore-loop domain, permeable to K^+^ and Na^+^ (Na^+^/K^+^ symport) (**Figure 3**). The HKT2 transporters help cereal crops take up K^+^ under salt stress (Horie et al., 2007; Horie et al., 2009; Ismail and Horie, 2017). Some reports suggest that dicot plant species have fewer HKT genes, belonging mainly to the HKT sub-class-I family. In contrast, monocot plant species have several HKT genes from both sub-gene families. For example, TmHKT1;4, TmHKT1;5, and OsHKT1;4, OsHKT1;5 in wheat belong to sub-class I family and mediate Na^+^ retrieval from xylem loading to restrict Na^+^ transport from roots to shoots or from leaf sheaths to leaf blades (Cotsaftis et al., 2012; Byrt et al., 2014; Ali et al., 2016; Suzuki et al., 2016).

It seems likely that the major net influx of Na^+^ is not controlled by a single transport protein but by several transport proteins. However, Na^+^/H^+^ antiporters and HKT1 transport proteins might determine net Na^+^ uptake and transport from roots to shoots of salt-stressed plants.

#### 3.2.3 Salt stress proteins involved in water balance

Water balance in crops under salt stress is an adaptive response, with the proteins associated with uptake and maintenance of water homeostasis substantially altered (Li et al., 2017d; McGaughey et al., 2018). Water uptake is regulated mainly by non-selective cation channels (NSCCs), i.e., water transport proteins or aquaporins (Byrt et al., 2017; Kourghi et al., 2017; McGaughey et al., 2018). Aquaporins include seven sub-families; the four main sub-families are plasma membrane intrinsic proteins (PIPs), tonoplast intrinsic proteins (TIPs), NOD26-like intrinsic proteins (NIPs), and small basic intrinsic proteins (Maurel et al., 2015; Maurel et al., 2016). Aquaporins play a significant role in passive bidirectional selective transport of small molecules, including water, CO_2_, salicylic acid, boron, urea, silicon, H_2_O_2_, and sodium ions (Byrt et al., 2017; Kourghi et al., 2017; McGaughey et al., 2018). Recent studies and some comprehensive reviews suggest that aquaporins are involved in maintaining water homeostasis in plants and play crucial roles in abiotic stress tolerance, stress signaling, nutrient uptake, transpiration, photosynthesis, and plant development (Maurel et al., 2016; Chaumont and Tyerman, 2017; McGaughey et al., 2018). For example, Qin et al. (2019) found that several aquaporins (PIPs) in halophyte *Eutrema salsugineum* had greater expression in roots than shoots under salt stress, consistent with root and leaf hydraulic conductivity. Moreover, they found that this differential expression in plant organs improved root water transport but decreased leaf transpiration under salt stress. Similarly, overexpression of plasma membrane or tonoplast intrinsic membrane aquaporins in rice (Katsuhara et al., 2003), tobacco (Aharon et al., 2003), and *Arabidopsis thaliana* (Wang et al., 2011) increased dehydration and salt stress sensitivity. More recently, Li et al. (2017d) reported that Na^+^ pretreatment to white clover plants enhanced aquaporin-mediated water transport from roots to leaves, improving stress tolerance. Overexpression of aquaporin genes from *Medicago sativa* increased salt tolerance in *Arabidopsis thaliana* by maintaining a higher K^+^/Na^+^ ratio and upregulating genes related to ABA and cytokinin and proline metabolism (Li et al., 2019). Regulation of aquaporin expression under salt stress depends on the intensity and duration of salt stress, plant organ, developmental stage, and species involved. For example, Arabidopsis plants subjected to 140 mM NaCl exhibited increased expression of most aquaporin genes (Geng et al., 2013). In contrast, Lee and Zwiazek (2015) reported that three-week-old Arabidopsis seedlings exposed to 10 mM NaCl for 1 h had decreased expression of all aquaporin genes except AtPIP2;6, whose expression did not change. Likewise, Kayum et al. (2017) found that 24 h of 200 mM NaCl increased the expression of BrPIP genes in *Brassica rapa*, but decreased the expression of *BrPIP1;3a/b*, *BrPIP2;7b/c*, and *BrPIP2;4a/b* beyond 24 h. In another study, 100 mM NaCl increased the expression of aquaporins (PIPs and TIPs) after 2–48 h in maize roots, whereas 200 mM NaCl decreased their expression after 24 h. Regulation of aquaporins under salt stress also occurs at the post-translational level by phosphorylation, glycosylation, methylation, ubiquitination, and deamination, affecting the opening and closing of aquaporins (Byrt et al., 2017; Kourghi et al., 2017; McGaughey et al., 2018). Moreover, several studies have demonstrated that signalling molecules and secondary messengers, such as pH, Ca^2+^ and H_2_O_2_ signals, regulate the opening and closing of aquaporins in response to salt stress (Bellati et al., 2010; Wudick et al., 2015; Bellati et al., 2016; Rodrigues et al., 2017). In sum, aquaporins regulate root hydraulic conductivity and water transport in plants under salt stress through two to three layers of translational and post-translational regulatory factors.

### 3.3 Proteins involved in osmoregulation (e.g., osmotin and many small-sized soluble proteins) for maintaining cell water content under saline stress

Several plant species accumulate compatible solutes and osmolytes, such as glycinebetaine, proline and trehalose, under salt stress with significant roles in osmotic adjustment, osmo-protection of cellular membranes and enzymes, and ROS detoxification (Ashraf and Foolad, 2007). In addition, several stress-related proteins are biosynthesized that play a vital role in plant adaptation to salt stress, acting as nitrogen storage and re-used during recovery from salt stress. Several years back, a salt-induced 26 kDa protein in tobacco was characterized and named as osmotin (Singh et al., 1987). Osmotin facilitates osmotolerance in plants (Barthakur et al., 2001) and modulates metabolism for osmotic adjustment (Raghothama et al., 1993). Thomas and Bohnert (1993) found greater accumulation of osmotin-like proteins (OLPs) in a halophyte *Mesembryanthemum crystallinum* under salt stress. OLPs (24–26 kDa) also help plants maintain cellular osmolarity by compartmentalizing solutes or through structural and metabolic alterations (Choi et al., 2013; Weber et al., 2014; Chowdhury et al., 2017). While osmotin and OLP-mediated salt tolerance occur in different crop species by improving plant water status and photosynthetic activity and reducing oxidative damage (Barthakur et al., 2001; Kumar et al., 2016; Hakim et al., 2018), the detailed molecular mechanism involved remains unexplored. In another study with halophyte Chenopodium quinoa Rasouli et al. (2021) found increase in 50, 13 and 8-fold increase in expression of 29B, osmotin like protein (OSML13) and dehydrin early responsive protein (ERD14), respectively. While assessing salt stress specific proteins in rice, López-Cristoffanini et al. (2021) found over expression of dehydrins in leaves that may play a key role in salt tolerance in rice.

Hurkman et al. (1991) found another group of proteins, germin-like 26 kDa proteins, in salt-stressed barley roots. The germins protein family has diverse roles in plants, including seed germination and ROS scavenging due to the presence of certain biochemical characteristics such as homo-polymer formation, cell wall localization, different enzyme activities, and glycosylation (Nakata et al., 2002; Davidson et al., 2009; Banerjee et al., 2017). Germins have the germin motif, which helps develop a jellyroll β-barrel structure (Caliskan, 2011). Germin-like proteins were upregulated in salt-stressed barley roots but downregulated in the coleoptile (Hurkman and Tanaka, 1996). Similarly, salt stress increased the biosynthesis of germins (GLP9, At4g14630; oxalate oxidase-like proteins) in *Arabidopsis thaliana* (Jiang et al., 2007). Several other proteomic studies on different plant species under salt stress suggest that the upregulation of germins or germin-like proteins involves cell wall cross-linking to maintain cellular anatomy and ROS-scavenging activity in the apoplastic region, e.g., barley (Ozturk et al., 2002; Fatehi et al., 2012), wheat (Kamal et al., 2012), rice (Banerjee et al., 2017; Frukh et al., 2020), spinach (Bai et al., 2014), and grapes (Cramer et al., 2007). In contrast, germin biosynthesis decreased in the roots of halophytic *Mesembryanthemum crystallinum* (Michalowski and Bohnert, 1992). Thus, osmotin, OLPs, germins, and germin-like proteins likely protect plants from salt-induced dehydration stress through osmotic adjustment in the leaves or modifying root cell walls to protect them from dehydration. However, changes in the expression of these proteins in plants under salt stress depend on the plant species and developmental stage.

### 3.4 Stress proteins involved in the maintenance of ultrastructure

Salinity stress causes osmotic stress by reducing water availability and thus inhibiting plant growth. During this process, salt stress poses substantial mechanical stress on plant cells by increasing the threshold of pressure for cell walls in expanding cells in root and stem meristematic tissues (Neumann, 2011). Such mechanical stress changes various cytoskeletal proteins, such as tubulin, actin, and kinesins, which help plants adapt to salt stress (Cheng et al., 2009; Barkla et al., 2013a; Kosova et al., 2013). For example, in rice roots, salt stress increased the cytoskeletal-cell wall linker proteins, which bind callose synthase in the plasma membrane and remodel cell wall properties of root cells (Cheng et al., 2009). Cell wall properties can be remodelled by various proteins with enzyme activities, such as glycosyl hydrolase family proteins, glycine-rich proteins, germins and germin-like proteins, and extensins (Bray, 2004; Jiang et al., 2007; Taiz et al., 2015). The abundance of proteins β-glucosidases and β-1,3-glucanase from the glucosyl hydrolase family increased in salt-stressed *Arabidopsis thaliana* roots (Jiang et al., 2007). In creeping bentgrass, increased β-D-glucan exohydrolase was associated with enhanced cell wall plasticity under saline conditions (Xu et al., 2010). Similarly, glycine-rich proteins with more than 60% glycine residues are important structural components of cell walls, with vital roles in plant resistance mechanisms against abiotic stresses, including salt stress (Mousavi and Hotta, 2005; Jiang et al., 2007). For example, salinity stress increased cell wall associated glycine-rich proteins associated with mechanical and defense properties of rice (Dooki et al., 2006) and cucumber (Du et al., 2010). Similarly, salt stress increased the expression of cellulose synthase in wild halophytic rice *Porteresia coarctata* (Sengupta and Majumder, 2009). In contrast, the cell wall elongation enzyme, xyloglucan endotransglycosylase, decreased in grapevine cultivar Chardonnay under salt stress and was associated with reduced growth (Vincent et al., 2007). These reports suggest that plant adaptations to salt stress require cytoskeletal proteins and plasma membrane associated proteins to improve cell wall mechanical properties under stressful environments.

### 3.5 Proteins involved in the protection of biomolecules such as LEAs and HSPs

Salt stress causes osmotic stress, i.e., water depletion in living cells resulting in cellular dehydration (Munns, 2002) and thus damaging macromolecular and cellular structures (Hoekstra et al., 2001). Osmotic stress tolerance in organisms requires several enzymes and biomolecules to prevent oxidative damage and maintain the native structure of enzymes, macromolecules, and membranes (Hasegawa et al., 2000; Maggio et al., 2002; Maggio et al., 2006; Shoji et al., 2006). Under normal conditions, macromolecules and membranes are hydrated, but under salt-induced osmotic stress, plants accumulate non-reducing oligosaccharides, compatible solutes, osmoprotectants, and osmoprotective proteins as a water replacement to protect macromolecular structures (Mansour, 1998; Hasegawa et al., 2000; Hoekstra et al., 2001; Ashraf and Foolad, 2007; Mansour and Ali, 2017). Osmo-protective proteins are hydrophilic proteins that include late embryogenesis abundant (LEA) proteins and HSPs (Rontein et al., 2002; Bhardwaj et al., 2013; Jacob et al., 2017). The extent of accumulation of these hydrophilic proteins is consistent with the degree of plant salt tolerance (Barkla et al., 2013a; Kosova et al., 2013; Liang et al., 2013). LEA proteins are categorized into seven subgroups based on sequence similarity and amino acid composition. The group 2 LEA proteins are referred to as dehydrins. LEA proteins protect proteins and membranes by replacing water, scavenging ROS, and binding with ions (Battaglia et al., 2008). Overexpression of LEA proteins enhances salt tolerance in various plant species, including Arabidopsis (Brini et al., 2007; Zhang et al., 2012b; Jia et al., 2014), rice (Hu et al., 2016), tomato (Muñoz-Mayor et al., 2012) and *Salsa crassa* (Yildiz and Terzi, 2021).

Although the detailed mechanism of action of LEA proteins is not known, we present a brief summary of current research here. The LEA proteins are unstructured in a hydrated state and known as intrinsically unstructured or disordered proteins (Tompa, 2005; Tompa and Kovacs, 2010; Pauwels et al., 2017). The intrinsically unstructured or disordered nature of LEA proteins is reportedly due to their high proportion of glycine and glutamine residues. In addition, LEA proteins possess unique characteristics, including excellent hydration potential, amplified speed of interaction with other proteins to support folding, and prevention of protein aggregation, i.e., they act as chaperones (Tompa and Kovacs, 2010; Liu et al., 2017), antioxidants (Hara et al., 2004), and cryoprotectants (Hughes and Graether, 2011). In the past decade, several reports and comprehensive reviews from Professor Peter Tompa’s group indicate that LEA proteins have several cellular regulatory functions in the cell cycle and transcription, translation, and post-translational modifications of LEA proteins, such as phosphorylation, methylation, and ubiquitination (Tompa, 2005; Tompa and Kovacs, 2010; Tompa and Han, 2012; Bhardwaj et al., 2013; Kovacs et al., 2013; Tompa et al., 2015; Tompa, 2016; Liu et al., 2017; Pauwels et al., 2017). Post-translational modifications change the folding characteristics of other proteins and their cellular localization (Liu et al., 2017). For example, phosphorylation of group-2 LEA proteins (dehydrin protein) was related to stress tolerance in *Craterostigma plantagineum* (Röhrig et al., 2006) and *Thellungiella salsuginea* (Rahman et al., 2011). Similarly, phosphorylation to 11-amino acid motif in group-3 LEA proteins enhanced salt and drought tolerance in *Escherichia coli* (Liu et al., 2010). More recently, phosphorylation at three sites of PM18 (group 3 LEA proteins) enhanced the protective effect in soybean cells under salt stress and a simulated dehydration state (Liu et al., 2017).

HSPs or chaperones are another group of powerful buffer proteins produced in plants that protect proteins from stress-induced misfolding (Carey et al., 2006; Swindell et al., 2007). In the past decade, this group of proteins has been studied extensively; it is now clear that the function of these proteins is not limited to protein folding, with a pivotal role in protein targeting and degradation to regulate signaling cascades under abiotic stresses, including salt stress (Al-Whaibi, 2011; Kadota and Shirasu, 2012; Kriechbaumer et al., 2012; Fu, 2014; Huang et al., 2014; Niforou et al., 2014; Jacob et al., 2017).

In summary, proteins initially discovered as membrane and macromolecule stabilizers have several physiological roles in stress tolerance and could be good targets for developing salt stress resistance in crop pants. However, more research is required to uncover their putative roles in stress tolerance.

### 3.6 Proteins involved in signal transduction

All land plants face continuously changing environments; thus, they require strategies to rapidly sense environmental changes such as increased salinity in the rhizosphere (Taiz et al., 2015). Plants should be able to detect multiple components of salt stress, such as toxic ions (Na^+^ and Cl^–^) in soil solution or plants, changes in osmotic potential in soil and plants, nutrient imbalance, and ROS generation (Shabala et al., 2015; Shabala et al., 2016). Sensing environmental changes and signaling to respond to these changes involves sensor proteins and response regulator proteins; signaling molecules such as secondary messengers play a role in signal amplifications and translation into a broad array of physiological alterations to optimize plant performance under salt stress (Taiz et al., 2015). Putative salt stress sensor proteins include plasma membrane localized ion transporter proteins, Na^+^ and K^+^ channels, mechanosensory proteins, H^+^-ATPases, and cytosolic Ca^2+^-binding proteins (e.g., calmodulin, calmodulin-like, calcineurin B like, and Ca^2+^-dependent protein kinases), and are comprehensively discussed elsewhere (Shabala et al., 2015). Overexpression or knock-down mutants of these proteins influence salt tolerance in plants (Wu et al., 1996; Liu and Zhu, 1998; Zhu et al., 1998; Zhu, 2000; Zhu, 2001a; Zhu, 2001b; Shi and Zhu, 2002; Shi et al., 2003; Gong et al., 2004; Chinnusamy et al., 2006; Maggio et al., 2006; Bai et al., 2013; Roy et al., 2013; Roy et al., 2014). The well-known signaling pathway for salt stress, i.e., SOS (salt overly sensitive pathway) and its components SOS3 (CBL, Ca^2+^-binding protein), SOS2 (CIPK24, calcineurin B like interacting protein kinase), SOS1 (Na^+^/H^+^ antiporters) are discussed at length in the salt exclusion section. However, other components include calcium sensor proteins, such as calmodulin and calmodulin-like proteins, detect changes in cytosolic Ca^2+^ under salt stress (Parre et al., 2007; Dodd et al., 2010; Laohavisit et al., 2013; Manishankar et al., 2018). These Ca^2+^-binding proteins or Ca^2+^-dependent protein kinases bind to calcium only under elevated calcium concentrations (Halfter et al., 2000; Kiegle et al., 2000; Dodd et al., 2010), executing downstream physiological responses or developmental changes (Charpentier and Oldroyd, 2013; Laohavisit et al., 2013; Choi et al., 2014; Manishankar et al., 2018) that are important in plant salinity tolerance, as all sensing mechanisms (channels, ATPases, mechano-sensors, etc.) operate in parallel and are integrated at the central calcium signaling hub (Charpentier and Oldroyd, 2013; Shabala et al., 2015; Manishankar et al., 2018). Recently, Chen et al. (2022) found major changes in expression of proteins related with ABA and GA metabolism and signaling pathways in maize under salt stress at the germination stage. Such kind of studies indicated that plant adjust hormonal homeostasis and signaling at the germination stage to adjust with salt stress. Such studies will definitely help in identification of master regulator in response to salt stress at the specific developmental stage. In sum, prominent salinity stress sensing mechanisms enable plants to decode information on the intensity, severity, and nature of salinity stress into stress-specific H_2_O_2_ and Ca^2+^ signatures to distinguish between osmotic and specific ion-toxic stresses. However, the sodium-specific sensor protein is not known, nor is information on tissue-specific sensing and signaling at different time scales (instantaneous hydraulic, slow hormonal).

### 3.6 Salt stress proteins involved in post-transcriptional and post-translational modifications

Expression of several genes related to defense, energy metabolism or photosynthesis are regulated by upstream transcriptional regulatory factors. In addition, appropriate folding and functioning of proteins require some more regulatory proteins at post-transcriptional, translational and post-translational level. For example, protein splicing factors or maturases help in plant group-II introns in self-splicing (Shevtsov-Tal et al., 2021). Salinity stress (200 mM NaCl) reduced the expression of maturase in alfalfa (Xiong et al., 2017). Down-regulation of maturase by salt stress indicated poor translation of proteins in shoots and roots of alfalfa. Expression of another protein with DNA chaperone activity and bind with single stranded DNA, nucleic acid binding protein (NABP), reduced in shoots and roots of alfalfa (Xiong et al., 2017). Nucleic acid binding protein is small and highly conserved protein across plant species. Like NABP, RNA-binding proteins (RBPs) also played vital role in cellular functions of protein transport and protein localization. RBPs play key roles in RNA splicing, mRNA stabilization, mRNA transport to targeted site and translation. Abundance of three RBPs has been observed in shoots and roots of salt stressed plants of alfalfa. These reports suggested that increase in abundance of nucleic acid binding proteins for RNA-post-transcriptional regulation is proposed to be important for improved salt tolerance in plants.

Ribosomes (ribonucleoprotein complex), translation initiation factors, elongation factors, and tRNA synthases are meant for protein translation, and help in post-translational modifications. Salt stress decreased the ribosomal proteins, while ribosomal components increased in *Arabidopsis thaliana* (Huang et al., 2018) and *Gossypium hirsutum* (Li et al., 2015) under salt stress. Likewise, 100 mM NaCl salinity caused upregulation of eukaryotic translation initiation factor (eIF 5A-2), while 200 mM NaCl salt stress down-regulated this ribonucleoprotein in alfalfa (Xiong et al., 2017). Similar down-regulation of expression of eIF 5A-2 due to salt stress has been observed in rice leaf lamina (Parker et al., 2006), and *SnRK2* transgenic rice (Nam et al., 2012). Proteins of other translation initiation factor (e.g., eIF3I) were down-regulated in the roots of salt stressed plants of *Arabidopsis thaliana* (Jiang et al., 2007) and *Gossypium hirsutum* (Li et al., 2015). Similarly, salt stress up-regulated eIF5A1 in salt tolerant cultivar of barley suggesting its role in general translation under salt stress and its contribution in salt tolerance (Mostek et al., 2015). While working with halophytes (*Suaeda maritima*, *Salicornia brachiata*) Benjamin et al. (2020) found up-regulation of eIF4A in *S. brachiata* under 200 mM NaCl salinity stress conditions, which indicated its involvement in salt tolerance of halophyte. Substantial increase in abundance of eIFs, EFs and ribosomal proteins in salt tolerant cultivar as compared to that in salt sensitive cultivar of rice (Frukh et al., 2020) and maize (Cheng et al., 2014). Protein translation effector (EF1B) regulates translation fidelity was up-regulated under salt stress in salt tolerant tomato accession than that in salt sensitive accession (Nveawiah-Yoho et al., 2013). Protein elongation factor (*GmEF4*) was up-regulated in salt tolerant soybean which is positively associated with it salt tolerance (Zhao et al., 2019). While working cotton Li et al. (2015) found increase in abundance of two ribosomal proteins and elongation factor under salt stress conditions. Based on these results, it is suggested that salt tolerant cultivars had better capability to maintain protein translation efficiency by regulating different components of translation machinery under salt stress conditions.

After protein synthesis, post-translational modifications such as phosphorylation, dephosphorylation, acetylation, methylation, glycosylation and ubiquitination are involved in correct folding, cellular localization, protein half-life, interaction with other proteins, cell signaling and fine-tuning of protein functioning (Krishnamurthy et al., 2018). It has been observed that salt stress increased the protein glycosylation and phosphorylation in canola Shokri-Gharelo and Noparvar, 2018). While comparative phosphor-proteomic analysis of salt tolerant and salt sensitive inbred lines of maize Zhao et al. (2019) reported that phosphoproteins associated with degree of salt tolerance in maize were up-regulated in salt tolerant inbred maize line. Kinases cause the phosphorylation of proteins to activate them and phosphatases dephosphorylate the proteins to de-activate the proteins. Çakır Aydemir et al. (2020) reported that salt stress changed the expression of 33 protein kinases and 16 phosphatases of grapevine rootstock. Post-translational events also include the appropriate folding of the proteins. Large number of proteins are meant for protein folding for their proper functioning and to avoid aggregation of nascent proteins. In addition, proteins with in appropriate folding or damaged during folding were detected and removed *via* 26S proteosome. Protein disulfide-isomerase A6 (PDI A6), a chaperone protein, helps inappropriate folded proteins in a correctly folded proteins without affecting disulfide shuffling (Gruber et al., 2006). Salt stress increased the abundance of PDI A6 proteins in rice (Nohzadeh et al., 2017) and cotton (Li et al., 2015). In contrast, 200 mM NaCl salinity stress down-regulated PDI A6 proteins in alfalfa (Xiong et al., 2017). As described earlier, post-translational modifications of proteins include ubiquitination to degrade the targeted negative-regulator proteins *via* 26S proteosome. Zhao et al. (2013) reported that salt stress increased the ubiquitination with subsequent degradation of targeted proteins and this plays a key role in maintaining level of proteins of key metabolic enzymes under salt stress. Similarly, salt stress increased the expression of protein post-translational modification regulator, transglutamase (TGase), in tobacco plants which improved the stability of chloroplastic proteins, photosynthetic capacity of tobacco plants and salt tolerance (Zhong et al., 2019). These reports suggested that regulation of salt stress proteins in different parts of different plant species is complicated in terms of translation and post-translational modifications but play a key role in salt tolerance.

### 3.7 Proteins involved in salt stress memory networks

**Figure 4:**
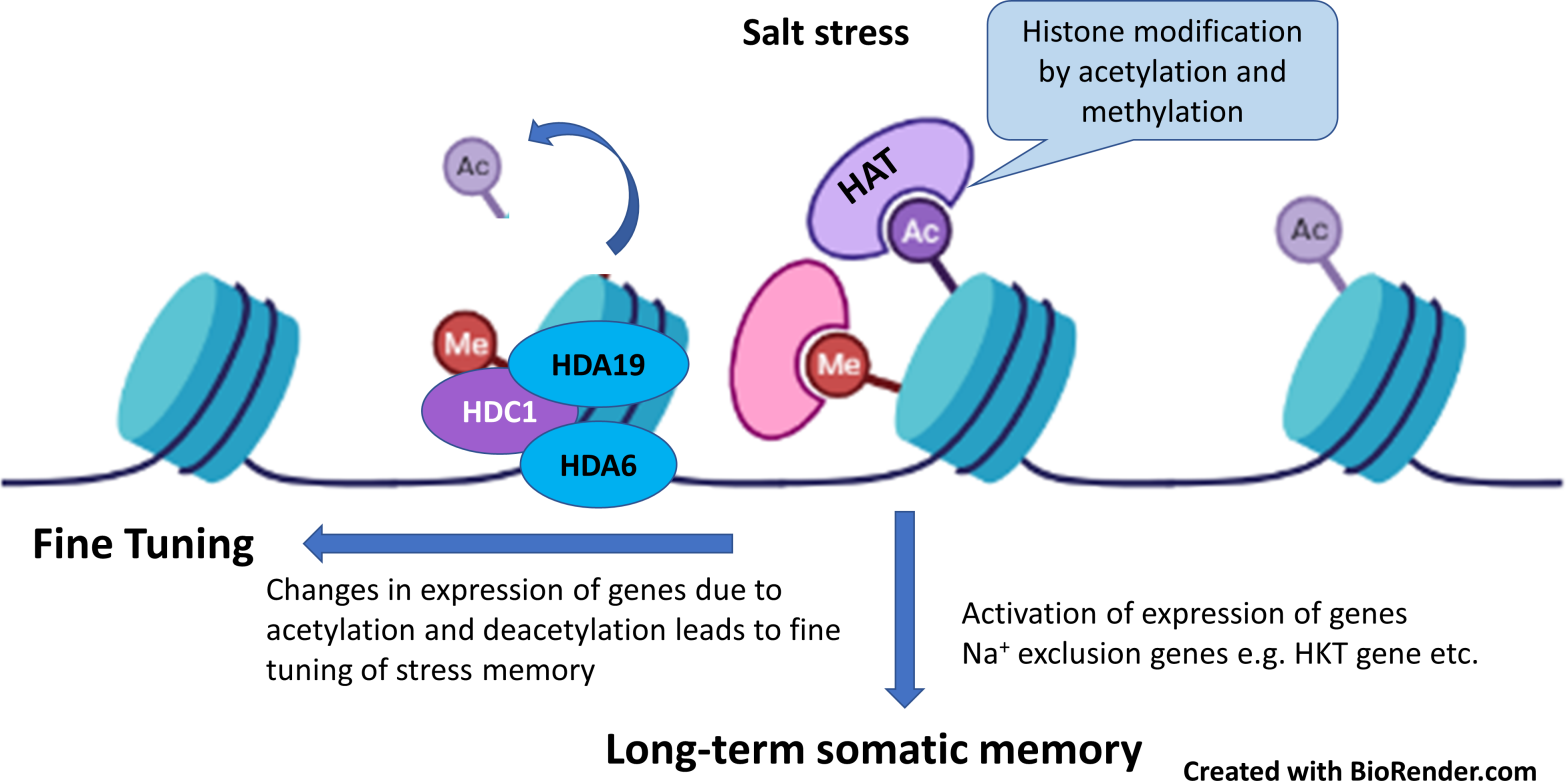
Salt stress modify the histone proteins by acetylation and methylation which activates the expression of genes such as HKT (high-affinity K^+^ channel) as long-term salt stress memory for rapid response to salt stress. In addition, histone acetylation and deacetylation played role in fine tuning of salt-stress memory (Adapted from Çakır Aydemir et al., 2020; Roy and Soni, 2022).

Plants save the stress events as molecular memory. The general mechanism of saving stress memory event occurs as over-accumulation of osmo-regulatory metabolites and phytohormones, synthesis of protective proteins, histone protein modification *via* DNA methylation and chromatin remodeling. Upon relieving from stress event, this stored memory initiates a quick and strong response for phenotypic variation of suitable traits that are responsible for long-term adaptation (Parejo-Farnés et al., 2019; Villagómez-Aranda et al., 2022). Stress memory can remain save over days, weeks or months in somatic cells (Mitotically stable) and it may remain stable and inheritable too (meiotically stable). Changes in gene expression by genome modification occurs through epigenetic marks and these include DNA methylation, histone protein post-transcriptional modification, and small RNAs (micro-RNAs, miRNA; small interfering RNA, siRNAs). Recently, while working with *Salvia miltiorrhiza* Yang et al. (2018) reported that proteins responsible for DNA-methylation (DNA methyl-transferases DNTM1, DNTM3A, DNTM3B) were over-expressed affected phenolic acid biosynthesis and stress tolerance. However, treatment with inhibitor of DNA methylation affected the gene expression of phenylpropanoid biosynthesis pathway and phenolic acid production (**Figure 4**). Similarly, in transgenic tomato for ERF1 (Ethylene response factor), DNA methylation at several genes initiate ethylene synthesis and signaling for stress tolerance (Zuo et al., 2018). Likewise, in another study with grapevine, salt stress reduced three-fold expression of 13 genes histone proteins (H2AX, H2A, H4, H3.2) (Çakır Aydemir et al., 2020). In contrast, salt stress activated global histone acetylation levels in maize genome by over-expression of histone acetyl-transferases (HATs), and these changes in histone protein modifications and DNA methylations were positively correlated with salt tolerance in maize (Li et al., 2014). Application of 500 mM NaCl cause the over-accumulation of histone H2A in halophyte *Salicornia brachiata* and its greater accumulation was positively associated with appropriate assembly of centromeres of chromosome, changes in gene expression and repair of DNA breaks. These reports suggested that establishing and resetting stress memory during plant life cycle is driven by post-transcriptional gene silencing *via* DNA-methylation or histone protein modifications that can be non-inheritable or inheritable. Since very few reports are available on this aspect, further research is need on this topic.

## 4 Mechanics and supra molecular kinetics of salt-stress proteins

Proteins perform a variety of functions in different parts of cell that include sensing and signaling, metabolism, transport of mineral nutrient and biomolecules, and transfer of genetic information (Nelson and Cox, 2021). Majority of proteins functions as homo-oligomer or hetero-oligomer assemblies. Proteins form tertiary structure by forming folding of globular domains consisting α helices, β-sheets and random coils. Proteins have two or more than two domains that are connected with coils or joints (Onck, 2017). These domains have ligand binding domains or other domains for various functions. Sometime ligand binding domain is present within several domains and other domains facilitate the ligand in interaction with ligand binding domain (Nelson and Cox, 2021). It is widely accepted that appropriate 3-D structure of the protein is responsible for proper functioning. However, any biotic or abiotic stress cause the changes in 3-D structure of protein from biologically active state to biologically inactive or biologically ill-function form. Changes in 3-D structure of proteins due to physical factors such as heat stress, salt stress etc. include domain unfolding, domain deformation, domain hinge motion and domain sliding motion. Domain hinge motion is very low deformation protein and mainly it consists of domain motion around a flexible hinge and it occurs in loops that joins α-helices or β-sheets (Nelson and Cox, 2021). All these changes in 3-D structure of proteins resulted in changed downstream biochemical, physiological and phenotypic effects on plants.

Of various salt stress proteins, high affinity potassium channel (HKT) has been studied in more detail in the mechanism of salt exclusions and salt tolerance. Based on transport and amino acid sequences, HKT family has been divided into two groups. The selective pore or selectivity filter in class 1 has Ser-Gly-Gly-Gly, whereas members of class 2 have selective pore with Gly-Gly-Gly-Gly. Four amino acids are placed on top of the four short loops or P-loop and make it as selectivity filter (**Figure 3**). The selective filters transport the Na^+^ ion selectively from xylem vessel to xylem parenchyma cells as xylem-Na-unloading process.

**Figure 5:**
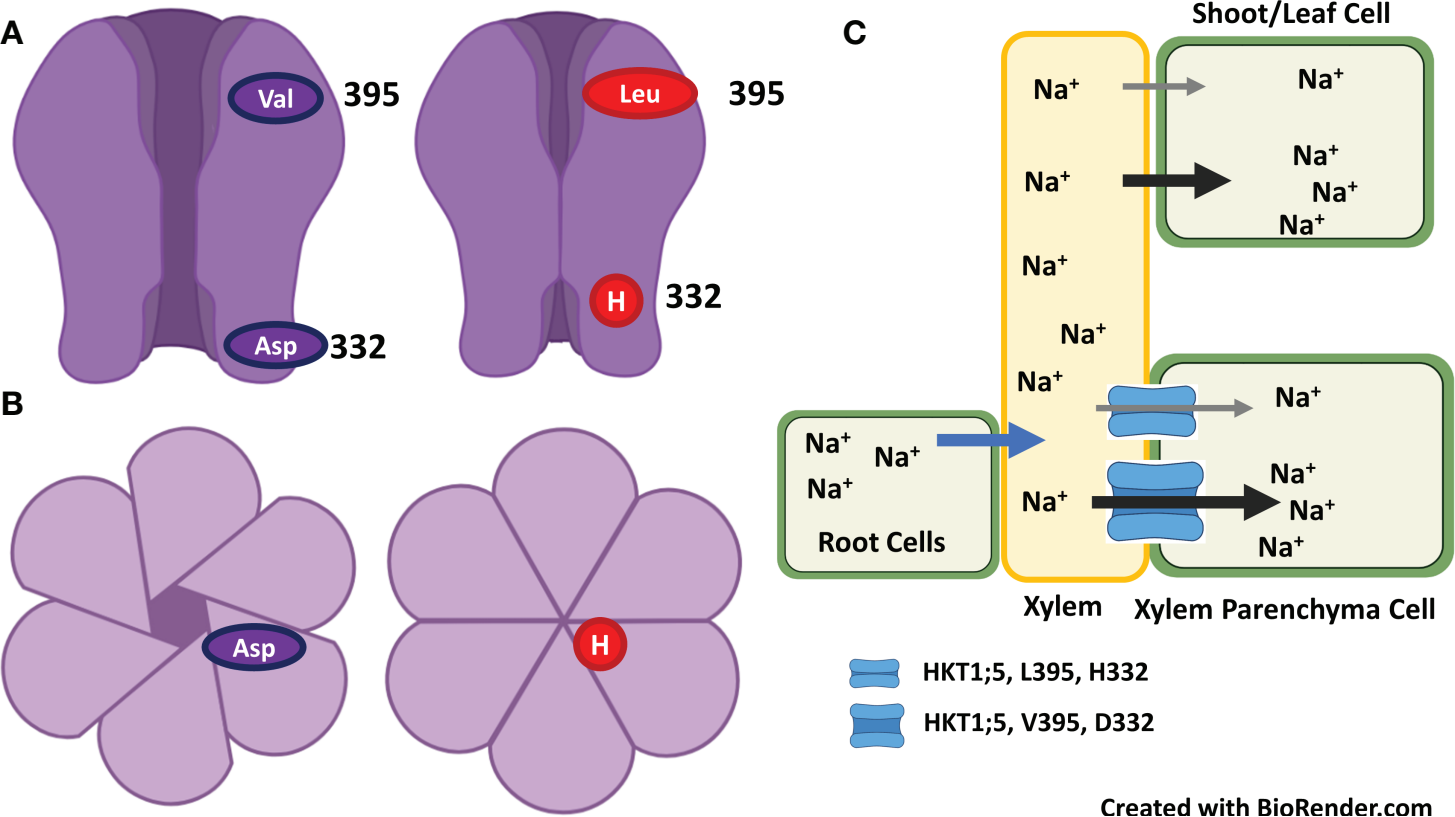
Na^+^ and/K^+^ selectivity for transport through HKT channel is determined by selective pores and aspartate at 332 and valine at 395 position which lie on the opening ends of channel but at the opposite sides. **(A)** Replacement of aspartate with histidine at position 332 that lies at the constriction of channel opening make Na^+^ passage difficult for entry – inhibit the Na^+^ exclusion. **(B)**. Surface view of HKT channel as open or closed for Na^+^ entry. **(C)**. Saline growth medium favors the entry of Na^+^ into root and then transport Na^+^ to leaves/shoots *via* xylem. HKT channel restrict the Na^+^ transport from root to shoot by excluding Na^+^ from xylem vessel to xylem parenchyma cells. Presence of valine at 395 and aspartate at 332 enhance the Na^+^ exclusion from xylem to xylem-parenchyma and thus induces salt tolerance, whereas presence of leucine at 395 and histidine at 332 reduce the Na^+^ exclusion and thus induces salt sensitivity (Adapted from Shohan et al., 2019; Pulipati et al., 2022).

The position of serine and glycine in selective pore is highly important in its ion transport capacity. For example, presence of serine favors the transport of Na^+^ only, whereas presence of glycine facilitates the transport of both K^+^ and Na^+^ ions. However, some studies have shown that HKT protein from class one has ability to transport of both K^+^ and Na^+^ ions in opposite directions. Similarly, TmHKT1;5 and TaHKT1;5 improved the salt tolerance by increasing Na^+^ exclusion. It has been observed that class 1 HKT channel played role in maintenance of K^+^, and Na^+^ unloading from root xylem parenchyma cells (Xu et al., 2018). In addition, class 1 HKT channel played role in salt tolerance of salt tolerant cultivar but not in salt sensitive cultivar. These results indicated that Na^+^ and/K^+^ selectivity for transport through HKT channel is not determined by selective pore alone, but it requires functioning of some other protein motifs as it determines the ion-transport capacity. Sequence comparison and amino-acid sequence of protein from both cultivars indicated variation in amino acid sequence at 140 (P/A/T/I), 184 (H/R), 332 (D/H) and at position 395 (V/L). Two amino acid variation occur in loop region between transmembrane domain 2 and 3 (TMD2 and TMD3), one in between TMD4 and TMD5 and one in between TMD5 and TMD6. While assessing association of amino acid substitution in class 1 HKT gene with salt tolerance in rice genotypes Shohan et al. (2019) found that amino acid substitution at position 140 (P/A/T/I) and 184 (R/H) were present in both salt tolerant and salt sensitive cultivars and thus these are not conserved in the context salt tolerance. In addition, aspartate at 332 and valine at 395 position is highly conserved in salt tolerant rice and wheat cultivars (Shohan et al., 2019). Three-dimensional structural analysis of HKT1;5 showed that both these amino acids (aspratic acid at 332 and valine at 395) lie on the opening ends of channel but at the opposite sides. For example, valine is located at the opening of channel towards xylem vessel, whereas aspartic acid lies at the opening channel toward xylem parenchyma cell. Position of both these amino acids is very strategic in controlling K^+^/Na^+^ transport and salt tolerance in plants. For example, histidine at position 332 lies in vicinity of large extra-cellular loop and at constriction of channel opening, which make Na^+^ passage difficult for entry in salt sensitive cultivar (**Figure 5**). However, aspartate make the extra cellular loop more dynamic and away from the channel opening thus it is helpful in Na^+^ efflux under normal or salt stress conditions (Shohan et al., 2019; Pulipati et al., 2022).

## 5 Conclusion and prospects

This review discussed various proteins involved in the complex salt tolerance mechanism of plants, with functions ranging from metabolism to ion homeostasis, maintaining water status to protect cellular structures, ROS scavenging to integrate signals at ROS and Ca^2+^ signaling hubs, and modulating physiological responses for developmental changes. Based on review of these studies, several promising protein indicators for salt tolerance are suggested, which include photosynthetic proteins (Psb27, LHCs, D2, rubisco, rubisco activase, sedo-heptulose-1,7 bisphosphatase), antioxidant enzymes (Cu/Zn-superoxide dismutase, peroxidase, glutathione reductase, catalase), mitochondrial and cellular respiration related proteins (glyceraldehyde-3-phosphate dehydrogenase, phospho-fructokinase, NAD(P)H-quinone oxido-reductase), ion transport related proteins (NHX, HKT1, HKT2, NRT1, NRT2), water transport related proteins (aquaporins, PIPs, NIPs), osmotic adjustment related proteins (P5CS, BADH, osmotin like protein, OSML13; dehydrin early responsive protein, ERD14; germins and germin like proteins, GLP9 etc.), cell wall modifications related (CCOMT, Extensins, expansins), salt stress sensors (RLKs, CaM, CBL, CIPKs) and transcription factors (MYB, NAC, WRKY, SnRK, bZIP). Several studies of comparative proteome profiling have shown abundance of salt stress proteins in halophytes or salt tolerant genotypes of same species under salt stress, which indicate their strong contribution in plant salt tolerance. Genes of these candidate protein markers can be utilized for the development of transgenics to improve salt tolerance in crops. A very few examples have been found about development of salt tolerant transgenics by overexpression of genes of these candidate proteins. Despite a Web of Science search on proteome profiling under salt stress revealing more than 2,000 reports, few success stories are available (Mansour and Hassan, 2022). Possible reasons for such poor success include the lack of interest in translating research into goods and services due to impecunious and improper research policies in universities (Gilliham et al., 2017), serious doubts for gene discovery research and experimental flaws (Flowers, 2004; Blum, 2014; Ashraf and Munns, 2022), and a misunderstanding that a single gene product will yield a desirable degree of salt tolerance (Flowers, 2004; Ashraf and Foolad, 2013; Shabala et al., 2016; De Costa, 2018). Molecular marker-assisted selection in breeding programs and pyramiding genes have been recently advocated for developing salt tolerance in crops (Ashraf and Foolad, 2013; Mujeeb-Kazi et al., 2019; Ashraf and Munns, 2022). However, some technological limitations (only a few genes can be transferred) have made it difficult to develop salt-tolerant genotypes using this approach (Shabala et al., 2016). This review discusses several salt stress proteins, only a few salt-stress proteins have gained attention for breeders in developing salt tolerance, including H^+^-ATPase, H^+^-PPase, Na^+^ and K^+^ transporters, and some regulatory proteins of these systems. Identifying proteins responsible for salt stress sensing will offer insights into understanding their functions in salt tolerance mechanisms. Nevertheless, understanding the detailed biochemical modes of action and physiological roles of different proteins related to metabolism, sensing and signaling, antioxidants, and membrane stabilizers may add a new dimension to programs aimed at breeding salt stress tolerant crops.

## Author contributions

Conceptualization, MA, FZ, and HRA; first draft preparation, MA, FZ, LZ, NA, ZUZ, and HRA; final draft preparation, review and editing, HRA, AM, MA, MSI, HMK, MAH, AES and KMHS. All authors contributed to the article and approved the submitted version.
